# The costs per live birth after uterus transplantation: results of the Swedish live donor trial

**DOI:** 10.1093/humrep/deae272

**Published:** 2024-12-15

**Authors:** Mats Brännström, Jana Ekberg, Lars Sandman, Thomas Davidson

**Affiliations:** Department of Obstetrics and Gynecology, Institute of Clinical Science, Sahlgrenska Academy, University of Gothenburg, Göteborg, Sweden; Stockholm IVF-EUGIN, Stockholm, Sweden; Department of Transplantation, Institute of Clinical Science, Sahlgrenska Academy, University of Gothenburg, Göteborg, Sweden; Department of Health, Medicine and Caring Sciences, Linköping University, Linköping, Sweden; Department of Health, Medicine and Caring Sciences, Linköping University, Linköping, Sweden

**Keywords:** uterus, infertility, transplantation, live birth, cost, health economic evaluation, pregnancy, *in vitro* fertilization, surgery, immunosuppression

## Abstract

**STUDY QUESTION:**

What is the cost per live birth after live donor uterus transplantation in a Swedish clinical trial setting?

**SUMMARY ANSWER:**

The total cost per child, from a health care perspective, was calculated to be €124 894 and if only surgically successful transplants are considered, the total cost per live birth was €107 120.

**WHAT IS KNOWN ALREADY:**

Uterus transplantation has proved to be a feasible treatment for uterine factor infertility by accomplished live births, both after live donor and deceased donor transplantation procedures. Our previous study, the only existing cost analysis of uterus transplantation, found that the initial (up to 2 months after surgeries) societal costs of preoperative interventions, live donor uterus transplantation surgeries, and postoperative care were between €50 000 and €100 000 (mean €74 000) in Year 2020 values per uterus transplantation. That study also included costs of sick leave for both donors and recipients.

**STUDY DESIGN, SIZE, DURATION:**

This real-data health economic cost study is based on a prospective cohort study, which included nine live donor uterus transplantation procedures. Study duration included the time from the first pre-transplantation investigation until postoperative controls after graft removal.

**PARTICIPANTS/MATERIALS, SETTING, METHODS:**

Recipients, live donors, and neonates of nine uterus transplantation procedures participated. The recipients and donors underwent pre-transplantation investigations with imaging, laboratory tests, and psychological/medical screening. In vitro fertilization with embryo cryopreservation was performed in advance of transplantation. Donor hysterectomy and transplantation were by laparotomy and the recipient received immunosuppression. Pregnancy attempts by ET started 1 year after transplantation and delivery was by caesarean section. Hysterectomy was performed either after birth of one or two children, after graft failure, or after multiple pregnancy failures. Nine transplantation procedures resulted in seven surgically successful (adequate blood flow and regular menstruations) grafts and six women delivered a total of nine children.

**MAIN RESULTS AND THE ROLE OF CHANCE:**

The total cost of preoperative investigations, live donor uterus transplantation, postoperative care, immunosuppression, IVF, follow-up, pregnancy care, delivery, and graft removal after completed childbirth(s) or failure to achieve live birth was calculated, based on inclusion of cost for six women, giving birth to a total of nine children, and three women, with no childbirth. Cost for live donors was also included in the analysis. The total cost per child was calculated to be €124 894. However, if only surgical successful transplants (seven out of nine transplants) are considered, the cost per live birth was €107 120. The cost for preoperative preparations with IVF, surgeries, and postoperative follow-up during the initial 2 months was around 53% of total costs. Smaller sub-costs were those for monitoring, ETs with additional IVF (14%), immunosuppression and other drugs from Month 3 until hysterectomy (13%), and pregnancy care with delivery and neonatal care (13%).

**LIMITATIONS, REASONS FOR CAUTION:**

Limitations are the restricted sample size, the experimental phase of the procedure and that the results only reflect the cost in one country (Sweden).

**WIDER IMPLICATIONS OF THE FINDINGS:**

The results provide the first information concerning the cost per child of the uterus transplantation intervention. In the future, the cost per child will most likely decrease due to predicted increase in the rate of surgical success, decreased surgical durations, decreased graft duration to achieve live birth(s), and increased rate of transplantations giving not only one, but two or three singletons.

**STUDY FUNDING/COMPETING INTEREST(S):**

Funding was received from the Jane and Dan Olsson Foundation for Science, the Knut and Alice Wallenberg Foundation, the Swedish Research Council, and an ALF grant from the Swedish state under an agreement between the government and the county councils. There are no conflicts of interest for any of the authors.

**TRIAL REGISTRATION NUMBER:**

NCT01844362.

## Introduction

The first live birth after uterus transplantation (UTx) occurred in 2014 (Brannstrom *et al.*, 2015) and this was after a live donor (LD) procedure within the first clinical UTx trial, involving nine transplantations in Sweden (Brannstrom *et al.*, 2014). Three years later, the first live birth after deceased donor (DD) UTx took place in Brazil (Ejzenberg *et al.*, 2019). Up until today, more than 70 UTx procedures, resulting in more than 40 live births, have been published (Brannstrom *et al.*, 2023a). The majority of the UTx attempts have been LD transplantations, with initial use of laparotomy in both LD and recipient and in recent years, there has been a gradual introduction of robotic surgery for donor hysterectomy (Brannstrom *et al.*, 2023b).

The conclusive success of a UTx procedure is marked by a live birth and therefore a cost analysis of UTx should take the actual live birth rate into account. Since a great majority of the UTx trials in the world are ongoing, with many uterus recipients not yet having completed their reproductive windows between the first embryo transfer (ET) and hysterectomy, the cumulative live birth rates of these trials are unknown.

The patients who would undergo UTx as an infertility treatment are women with absolute uterine factor infertility (AUFI), which is due to either a congenital/surgical absence of the uterus or the presence of a non-functional uterus, which cannot sustain a pregnancy for a duration that permits neonatal viability (Hur *et al.*, 2019). Based on results from a study in the UK, the AUFI condition affects around 1:500 women of fertile age (Sieunarine *et al.*, 2005). The Mayer–Rokitansky–Küster–Hauser syndrome (MRKH), which is a congenital Müllerian agenesis-related malformation (Herlin *et al.*, 2020), is the cause of AUFI in more than 95% of uterus recipients of the UTx procedures reported worldwide (Brannstrom *et al.*, 2023a,b) and in 8/9 patients of the present study.

UTx is now in a transition phase from an experimental to clinical procedure, with Germany being the first country to list UTx as an infertility treatment within a health insurance system (Brannstrom *et al.*, 2023b) and with the possibility in the USA for out-of-pocket-financed UTx (Polk *et al.*, 2022). One ethical concern around UTx is how this infertility treatment should be prioritized in relation to other healthcare interventions (Wilkinson and Williams, 2016). In that context, knowledge of the cost and the cost-effectiveness of UTx are of importance (Sandman, 2018), since the UTx infertility treatment would belong to a borderline category of possibly publicly/health insurance-funded interventions in a healthcare system. Thus, it is of great importance to estimate the costs involved for a live birth after UTx, with such calculation also involving costs for failed UTx procedures within a series.

To the best of our knowledge, there is solely one published real-data health economic analysis of UTx. This is our previous study (Davidson *et al.*, 2021) of the same cohort as the present study, but only including costs from preoperative investigations until 2 months after surgeries and thus with no possibility of relating the costs to live birth(s). Live births typically come from around 1.5 years to 6 years after UTx (Brannstrom *et al.*, 2023a). In our previous cost analysis, we found a mean health and societal cost per procedure of around €74 000 (Year 2020 values), and this also included costs of sick leave for LD and recipient (Davidson *et al.*, 2021).

The objective of the present study was to expand upon the initial study to evaluate the actual cost per live birth. This analysis could serve as a foundation for comparing UTx with other infertility interventions within the healthcare system and would provide valuable background data for conducting cost-effectiveness evaluations. Furthermore, such a cost analysis would provide an opportunity to identify areas for potential cost reductions in UTx.

## Materials and methods

### Patients and procedural map

The patients of the present study comprise the nine recipients and their directed LDs of the world’s first clinical UTx trial, with the recipients being eight women with MRKH and one woman hysterectomized for cervical cancer (Brannstrom *et al.*, 2014). The median (range) ages of recipients and the LDs at the time of surgeries were 34 (27–38) years and 53 (37–62) years, respectively. Out of the nine UTx procedures (UTxP1–UTxP9), seven were surgically successful and two resulted in early graft failures, as described in detail in the 6-month outcome report (Brannstrom *et al.*, 2014). Seven out of the nine recipients commenced pregnancy attempts by ETs from 12 months after UTx (Brannstrom *et al.*, 2022).

The procedural map of all interventions was according to a predetermined research protocol. Preoperative investigations and interventions included clinical chemistry and immunology blood tests, microbiology tests of vaginal/cervical samples, cytological analysis of vaginal/cervical smears, multi-modal imaging (Leonhardt *et al.*, 2022), psychological evaluations, standard medical and gynaecological investigations, as well as IVF with cryopreservation of at least 10 embryos of a mixture of cleavage stage embryos and blastocysts (Brannstrom *et al.*, 2014).

The nine LD UTx procedures resulted in nine children born to six mothers. Three of the transplants did not result in any live birth, with the causes being early graft failure in two procedures (UTxP2, UTxP9) and multiple implantation failure/repeated miscarriages in the recipient of UTxP3, as described in detail elsewhere (Brannstrom *et al.*, 2014, 2022). All nine transplants are included in the calculations. All UTx procedures were performed from September 2012 to April 2013 and the last graft was removed in January 2020.

### Calculation of costs

The data was analysed according to per protocol analysis, and the methodology of cost analysis was in accordance with Drummond *et al.* (2015). The CHEERS 2022 checklist (Husereau *et al.*, 2022) was used as guidance. All costs were identified, quantified in physical units, and then valued. The analysis was done from a healthcare perspective. The cost of resource use was determined based on its opportunity cost, i.e. the best alternative use of the resources. Costs were estimated in Swedish Krona (SEK) in Year 2024 values and presented in Euro (€) using an exchange rate of €1 = SEK11.34 (20 March 2024). Costs from our earlier estimations from Year 2020 (Davidson *et al.*, 2021) have been adjusted for inflation with 24% until March 2024.

In our earlier publication (Davidson *et al.*, 2021), the cost of UTx was calculated from the start of preoperative investigations until 2 months after surgeries. This calculation is now updated with the full costs per live birth, which include monitoring costs of the graft and recipients, immunosuppression, ETs, additional IVFs, interventions at complications of recipients and LDs, prenatal care during pregnancies, caesarean sections, postpartum and postnatal care, hysterectomies, and follow-up visits.

Most prices of tests and clinical procedures were taken from the tariff list Year 2024 of Sahlgrenska University Hospital, Gothenburg, Sweden (Västra Sjukvårdsregionen, 2024), which is the hospital where all procedures and monitoring took place. This price list is based on cost-per-patient data (including overhead costs), with an extra 2% overhead fee for treating patients from another health region in Sweden. The cost of graft hysterectomy was estimated in relation to surgical time since there was a large variation in durations and the surgical procedure took a much longer time than an ordinary hysterectomy, as described in detail elsewhere (Karlsson *et al.*, 2022). Every minute used for hysterectomy has a price of 34.3€ (Västra Sjukvårdsregionen, 2024), which includes costs for procedure, operation theatre staff, anaesthesiology staff, as well as for two surgeons. The costs for postnatal treatment of the infants include ordinary care (included in cost for caesarean section) for five neonates and additional costs for neonatal intensive care unit (NICU) for four neonates, cared for at NICU level II, according to the policy statement concerning grading of different levels of neonatal care of the American Academy of Pediatrics (American Academy of Pediatrics Committee on Fetus and Newborn, 2012). The lengths and causes of NICU care are specified elsewhere (Brannstrom *et al.*, 2022). The drug costs were taken from the official tariff list of 2024 (Apoteket, 2024) and were calculated per mg for most pharmaceuticals and estimated for antibiotics per week. All unit costs are presented in Table 1.

**Table 1.** Unit costs (€) for drugs and interventions used in the calculations.

	Cost (€)
Drugs	
Tacrolimus, 1 mg	2.0
Prednisolone, 2.5 mg	0.1
Azathioprine, 75 mg	0.2
Acetylic acid, 75 mg	0.1
Intravenous antibiotics (per week)	90
Oral antibiotics (per week)	10
Anti-thymocyte globulin, 25 mg	180
Methylprednisolone, 500 mg	40
Omeprazole, 20 mg	0.3
Ferric carboxymaltose, 10 ml (500 mg Fe)	178
Valganciclovir (450 mg)	8.5
Mycophenolate mofetil (250 mg)	0.5
Clinical chemistry analyses* and urine culture	112
Vaginal bacterial culture	46
Pathology—cervical biopsy	562
IVF with fresh ET	3306
Frozen ET	1479
Antenatal examination—obstetrician + ultrasound	265
Antenatal examination—midwife	147
Caesarean section	7299
Hospital stay—postoperative (per day)	422
Hospital stay—with intervention (per day)	450
NICU admission cost	1794
NICU cost per day	199
Graft hysterectomy (per minute)	34.3
Follow-up visit—gynaecologist	205
Counselling—psychologist	150

* Blood (haemoglobin, white blood cell count, platelets, sodium, potassium, creatinine, liver enzymes, tacrolimus concentration, glucose), and urine (albumin/creatinine ratio) clinical chemistry analyses.

NICU, neonatal intensive care unit.

## Results

### Initial costs

The early costs from the start of preoperative investigations until 2 months after surgeries of donor and recipient are given in Table 2. The donor costs were comparatively similar among the nine procedures, but with markedly higher cost in UTxP2, related to the long duration of donor hysterectomy. The initial cost for the recipient of UTxP2 was also the highest, rendering the initial total costs for that specific procedure being around 36% higher than the median total cost (€63 429).

**Table 2.** Early total costs (€) from baseline until 2 months after operation (including both donor and recipient).

UTxP	Donor cost	Recipient cost	Total cost
1	26 563	40 402	66 965
2	37 551	48 765	86 316
3	28 308	36 878	65 186
4	25 903	37 379	63 282
5	25 607	37 404	63 011
6	26 440	31 833	58 273
7	25 800	37 629	63 429
8	26 929	40 437	67 366
9	29 439	32 796	62 235
**Mean**	**28** **060**	**38** **169**	**66** **229**

Included are costs for screening preparations, IVF, anaesthesia, surgeries (hysterectomy and transplantation), postoperative hospitalization, postoperative tests, re-hospitalization, immunosuppression for 2 months, and other pharmaceuticals. UTxP refers to number, in chronological order, of uterus transplantation procedure. The bold values are the mean costs of donor, recipient and the total cost (donor+ recipient) of the nine procedures.

### Costs from month 3 until after graft hysterectomy

The costs of pharmaceuticals during the times with the grafts from start of postoperative Month 3 until hysterectomy, with a wide variation in durations, are shown in Table 3. These costs were low in recipient of UTxP2 since that graft was removed 3 months after UTx. The highest drug-specific cost was that of tacrolimus (Tac), which was the principal maintenance immunosuppression. Naturally, there was a relationship between time of exposure and the total costs for the drugs.

**Table 3.** Costs (€) of drugs for recipients of each uterus transplantation procedure (UTxP) from 2 months after operation until graft hysterectomy.

UTxP	Days of exposure	Tac costs	MMF costs	AZA costs	Pred costs	MPred costs	ATG costs	Ome costs	Valg costs	ASA costs	Other costs	Total cost
**1**	750 + 750	20 958	414	0	162	0	0	23	247	150	1068	**23** **022**
**2**	43	143	42	0	361	0	0	27	366	4	230	**1173**
**3**	2062	28 868	270	502	531	300	900	902	757	206	890	**34** **126**
**4**	1110 + 518	22 792	301	0	94	0	0	17	247	163	534	**24** **148**
**5**	603	8442	516	107	180	160	0	255	298	60	0	**10** **018**
**6**	1550 + 518	34 636	1036	620	315	120	0	1216	587	247	356	**39** **133**
**7**	962	13 468	464	253	204	0	0	142	281	96	0	**14** **908**
**8**	627	8778	462	0	220	0	0	154	264	63	0	**9941**
**9**	0	0	0	0	0	0	0	0	0	0	0	**0**
**Mean**	**1100**	**15** **343**	**389**	**165**	**230**	**64**	**100**	**304**	**339**	**110**	**342**	**17** **385**

The duration (days) of exposure for principal immunosuppression (tacrolimus) beyond 2 months, with separation for the first and second child are also given. The recipient of UTxP9 had graft removal within the first post-UTx week.

UTxP, uterus transplantation procedure number; Tac, tacrolimus; MMF, mycophenolate mofetil; AZA, azathioprine; Pred, prednisolone; MPred, methylprednisolone; ATG, anti-thymocyte globulin; Ome, omeprazole; Valg, valganciclovir; ASA, acetylic acid; Other, antibiotics and ferric carboxymaltose. The bold values at the bottom are means of the nine procedures. The bold values to the right are the total costs for all pharmaceuticals for each of the nine procedures.

In Table 4, the costs for all the major procedures from 2 months after operation, excluding costs for pharmaceuticals, are specified for each procedure. The highest cost was for recipient of UTxP6. She had the longest duration with the graft (6.9 years) and delivered two babies. She incurred significant expenses for IVF due to the necessity of using embryos created post-UTx. This arose from her divorce from her partner, after which she underwent IVF using donor sperm. The cost was relatively high for recipient UTxP3, who did not achieve a live birth despite going through 16 ETs (Brannstrom *et al.*, 2022). Due to the many pregnancy attempts, the costs for ETs were substantially higher than for any other patient and she also had the second highest cost for post-UTx IVF.

**Table 4.** Number of interventions and the corresponding cost from 2 months after uterus transplantation for nine procedures (UTxP).

	UTxP1		UTxP2		UTxP3		UTxP4		UTxP5		UTxP6		UTxP7		UTxP8		UTxP9	
	No.	Cost	No.	Cost	No.	Cost	No.	Cost	No.	Cost	No.	Cost	No.	Cost	No.	Cost	No.	Cost
Sampling and laboratory	1	112	1	112	1	112	1	112	1	112	1	112	1	112	1	112	0	0
Vaginal culture	8 + 11	874	0	0	11	506	20 + 12	1472	2	92	14 + 11	1150	3	138	2	92	0	0
Pathology	6 + 8	7868	0	0	21	11 802	7 + 3	5620	14	7868	10 + 13	12 926	13	7306	14	7868	0	0
IVF with ET	0 + 2	6612	0	0	3	9918	0 + 1	3306	0	0	3 + 1	13 224	0	0	0	0	0	0
Frozen ET	4 + 3	10 353	0	0	13	19 227	7 + 1	11 832	1	1479	4 + 0	5916	6	8874	1	1479	0	0
Ultrasound by obstetrician	6 + 6	3180	0	0	0	0	8 + 8	4240	5	1325	12 + 10	5830	5	1325	5	1325	0	0
Antenatal care visit	7 + 5	1764	0	0	0	0	7 + 7	2058	5	735	12 + 7	2793	5	735	5	735	0	0
Caesarean section	1 + 1	14 598	0	0	0	0	1 + 1	14 598	1	7299	1 + 1	14 598	1	7299	1	7299	0	0
Hospital stay caesarean section (days)	7 + 0	2954	0	0	0	0	6 + 0	2532	3	1266	3 + 0	1266	8	3376	6	2532	0	0
Graft hysterectomy (minutes)	175	6003	192	6586	152	5214	114	3910	266	9124	117	4013	167	5728	257	8815	0	0
Hospital stay—graft hysterectomy (days)	6	2532	2	844	3	1266	6	2532	3	1266	3	1266	5	2110	5	2110	0	0
Hospital stay—complications (days)	2	900	6	2700	11	4950	7	3150	0	0	0	0	0	0	0	0	0	0
Maternity ward for child (days)	2 + 6	1592	0	0	0	0	4 + 4	1592	35	6965	3 + 3	1194	4	796	12	2388	0	0
Neonatal intensive care (days)	7 + 0	3187	0	0	0	0	0 + 2	2192	7	3187	0 + 0	0	2	2192	5	3789	0	0
Follow-up meeting by obstetrician	1 + 1	410	0	0	0	0	0 + 1	205	2	410	1 + 1	410	1	205	1	205	0	0
Psychologist	1 + 0	224	0	0	3	672	0 + 0	0	0	0	1 + 0	224	0	0	0	0	0	0
**Events cost**		**63** **163**		**10** **242**		**53** **667**		**59** **351**		**41** **128**		**64** **922**		**40** **196**		**38** **749**		**0**

The number of interventions for the first and second child is separated by + for UTxP1, UTxP4, and UTxP6. The bold values at the bottom are the total costs for interventions, not including pharmaceuticals, for each procedure.

Treatments of children in NICU added substantial costs for four recipients.

### Total costs

**Figure 1.**
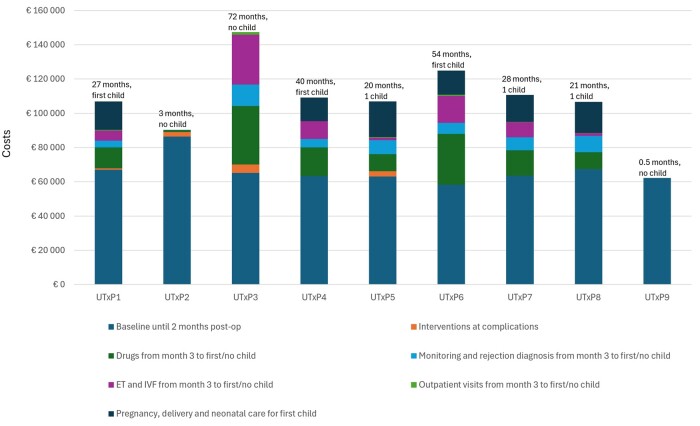
**Costs from baseline until first/no child for all nine uterus transplantation procedures (UTxP).** Duration (months) of the times from uterus transplantation to graft removal in procedures with no live birth (UTxP2, UTxP3, and UTxP9) and in other procedures to first child (UTxP1, UTxP3 and UTxP5, UTxP6, UTxP7, and UTxP8), are given on top of each bar.

A summary of the total costs for each patient, including the UTx-related surgeries and preoperative investigations with IVF (‘Baseline until 2 months postoperative’), is given in Table 5. The largest sub-costs were the initial costs of preoperative investigations with IVF and the two major surgeries (donor hysterectomy, transplantation) of each case. The total cost for the nine procedures, resulting in nine live births, was €1 124 043. The estimated cost per live birth (*n* = 9) would then be €124 894. When calculating costs per child for only the surgically successful grafts (i.e. omission of UTxP2 and UTxP9), the cost per child was €107 120. In Fig. 1 and Table 5, the total costs from baseline until first child/no child (graft failure before ET or never a live birth) are shown, providing a mean cost per UTxP at €107 208. The lowest such costs (€62 235; €90 301) were in two patients (UTxP2, UTxP9) with early graft failures and the highest such cost (€124 927) was in one patient with no child after multiple ETs, resulting in implantation failures or miscarriages. The additional costs for the second child (UTxP1, UTxP4, and UTxP6) were lower (mean €31 951).

**Table 5.** Total cost and sub-costs for each uterus transplantation procedure (UTxP), where three procedures did not result in any live birth (UTxP2, UTxP3, UTxP9), three procedures resulted in one live birth each (UTxP5, UTxP7, UTxP8), and three procedures resulted in two live births (UTxP1, UTxP4, UTxP6).

	UTxP1	UTxP2	UTxP3	UTxP4	UTxP5	UTxP6	UTxP7	UTxP8	UTxP9	Mean cost
Baseline until 2 months postoperative	66 965	86 316	65 186	63 282	63 011	58 273	63 429	67 366	62 235	66 229
Interventions at complications	900	2700	4950	0	3150	0	0	0	—	1463
Drugs from Month 3 to first/no child	12 252	1173	34 126	16 730	10 018	29 709	14 908	9941	—	16 107
Monitoring and rejection diagnosis from Month 3 to first/no child	3852	112	12 420	4972	8072	6376	7556	9551	—	6614
ET and IVF from Month 3 to first/no child	5916	—	29 145	10 353	1479	15 834	8874	1479	—	10 440
Outpatient visits from Month 3 to first/no child	429	—	1472	0	410	629	205	205	—	479
Pregnancy, delivery, and neonatal care for first child	16 461	—	—	13 776	20 777	14 106	15 723	18 068	—	16 485
**Total cost first/no child**	**106** **775**	**90** **301**	**147** **299**	**109** **113**	**106** **917**	**124** **927**	**110** **695**	**106** **610**	**62** **235**	**107** **208**
Drugs from first child to second child	10 770	—	—	7418	—	9424	—	—	—	9204
Monitoring and rejection diagnosis from first child to second child	5002	—	—	2232	—	7812	—	—	—	5015
ET and IVF from first child to second child	11 049	—	—	4785	—	3306	—	—	—	6380
Outpatient visits from first child to second child	205	—	—	205	—	205	—	—	—	205
Pregnancy, delivery from first child to second child	9224	—	—	12 640	—	11 575	—	—	—	11 146
**Total cost of second child**	**36** **250**	—	—	**27** **280**	—	**32** **322**	—	—	—	**31** **951**
Graft hysterectomy	8535	7430	6480	6442	10 390	5279	7838	10 925	—	7915
**Total cost**	**151** **560**	**97** **731**	**153** **779**	**142** **835**	**117** **307**	**162** **528**	**118** **533**	**117** **535**	**62** **235**	**124** **894**

The bold values in the middle of the table refer to total cost of the first child (6 patients; UTxP1, UTxP4, UTxP5, UTxP6, UTxP7; UTxP8), no child (3 patients; UTxP2, UTxP3, UTxP9), and in the right column the mean cost for all procedures is shown in bold. The bold values in the third line from the bottom refer to the total costs for the interventions and pharmaceuticals form birth and care of the first child until delivery and care for a second child for the three women who delivered two children (UTxP1, UTxP4, UTxP6), and in the right column the mean cost for these three procedures is shown in bold. The bold values in the bottom line refer to the total cost for interventions and pharmaceuticals for each procedure, and in the right column the mean cost for these three procedures is shown in bold.

## Discussion

### Main findings and background

The main finding of this first-ever estimation of costs per live birth in UTx found a total cost per child of €124 894. More than half of the costs were related to costs of preoperative investigations, including IVF, and surgeries with associated postoperative care. When calculating costs per child for only the surgically successful grafts, the cost per child was €107 120.

Cost estimations for novel medical procedures within the broader field of medicine, including interventions for infertility treatment, are essential. These estimations serve as crucial tools for discussions and assessments regarding affordability and prioritization. Importantly, a health economic analysis of the cost per child in women with uterine grafts can only be performed upon completion of a full case series within a clinical trial, spanning the entirety of all participating women’s reproductive window. This window extends from the first ET until the removal of the uterine graft. The reproductive window for women undergoing UTx has typically had a duration of 2–6 years, facilitating the possibility of one or two live births. The initial ET has usually been performed within 8–12 months after UTx (Brannstrom *et al.*, 2023a,b). The postponement of the first ET is to allow for some months of healing of anastomoses as well as for stabilization and lowering of immunosuppression. The reason to remove the graft after completed childbirth(s) and thereby to restrict the duration of the graft to a relatively short time is that the main component of immunosuppression, the calcineurin inhibitor, is nephrotoxic and medication for many years will substantially increase the risk of irreversible kidney damage (Ekberg *et al.*, 2023). Based on the well-known nephrotoxicity of calcineurin inhibitors, there is a general recommendation of removal of a uterine allograft within 7 years but in cases with no live birth despite multiple implantation failures, this time interval has been extended to up to 9 years (Ozkan *et al.*, 2022). Naturally, the chance of a live birth increases with a growing number of IVF treatments also for women after UTx, similar to an ordinary IVF population, where increased cumulative live birth rates were seen up to at least six IVF cycles with all their associated ETs (Malizia *et al.*, 2009).

### Comparison with cost estimation of uterus transplantation

There exists only one comparative study from another group, in the field of health economics of UTx. A feasibility study out of the Netherlands was conducted to estimate the cost of UTx in preparation for a possible start of this procedure in the country (Peters *et al.*, 2020). The cost per UTx procedure was estimated to nearly €100 000, but not including costs of pregnancy care and delivery, as they would be covered by medical insurance. Their estimation of summary cost of screening with IVF and LD UTx surgeries, by robotics in LD and laparotomy in recipient, was around €77 000. This is somewhat higher, but in the same range as the corresponding real costs for these procedures of the present study, with a mean of around €66 000. Importantly, the study from the Netherlands is not based on real-life data and presents a theoretical prediction of costs involved (Peters *et al.*, 2020). The latter study also assessed the interest of UTx as an infertility treatment for women with MRKH and found that a majority (60%) expressed a preference for UTx over gestational surrogacy (30%) and adoption (6%).

### Cost comparison between uterus transplantation and gestational surrogacy

Gestational surrogacy, although not legally permitted in most countries worldwide (Salama *et al.*, 2018), remains a traditional alternative to UTx to acquire motherhood for women with AUFI. However, the financial implications associated with commercial gestational surrogacy can be substantial. In the USA, a complete process of surrogacy may cost as much as $200 000, with sub-costs of $20 000–80 000 for medical expenses, $3000–15 000 for legal support, $6000–54 000 for surrogacy recruiting programmes, and $20 000–55 000 for carrier compensation (Brandao and Garrido, 2022). In low-income countries, the total expenses involved in a full surrogacy process typically amount to less than half of those incurred in the USA (Brandao and Garrido, 2022). When considering the combined expenses of a child following UTx in Sweden, as estimated in the current study, the costs are comparable to those associated with a child born through gestational surrogacy in a low-income country. However, the advantages of UTx include fewer ethical and legal concerns, as well as the future mother bearing the risks associated with pregnancy-related complications, such as gestational diabetes, venous thromboembolism, and hypertensive disorders of pregnancy.

### Costs of preoperative investigations/interventions and surgeries

The results of the present study reveal that around 53% of the overall costs of UTx with live birth are attributed to early expenses associated with preoperative investigations, including pre-UTx IVF, and the surgical procedures along with hospital care. This observation is not surprising given the extensive and time-consuming nature of the surgical procedures of LD UTx. The costs of these procedures were calculated based on the duration of anaesthesia and surgery, resulting in an average total cost per UTx procedure for anaesthesia, surgeries, and postoperative care of approximately €33 000 in Year 2020 values (Davidson *et al.*, 2021).

### IVF costs

As regards costs related to assisted reproduction in UTx of the present study, the majority of IVF stimulations with embryo cryopreservation were performed during the pre-UTx period in order to store at least 10 embryos, which was estimated to be enough for at least one live birth. However, four out of the seven patients with surgically successful grafts performed additional IVF cycles post-UTx and these IVF treatments resulted in five live births (Brannstrom *et al.*, 2022). Thus, most of the children were born from post-UTx IVF, indicating that these additional IVFs were cost-effective procedures, considering that preoperative investigations together with surgeries account for about 53% of the total costs.

The largest total post-UTx cost was in the recipient of UTxP6 who had four post-UTx cycles to achieve two live births, since she could not use the embryos cryopreserved before UTx. She separated from her partner shortly after UTx and subsequently went through four IVFs with donor sperms (Brannstrom *et al.*, 2022). It is important to highlight that recipient of UTxP3, who unfortunately never experienced a live birth, underwent as many as 16 ETs and three post-UTx IVF procedures. Naturally, the costs for the assisted reproduction for the whole cohort would have been reduced if the exceptional circumstances of recipient of UTxP3 had not arisen.

In the IVF procedures of the present study, a total of 35 Day 2 ETs and 11 blastocyst ETs were conducted to achieve the nine live births (Brannstrom *et al.*, 2022). Consequently, 37 ETs, each with a cost of €1479, resulting in an overall expenditure of nearly €55 000, did not yield a live birth. Naturally, employing an ET strategy exclusively utilizing blastocysts, known for their high pregnancy potential, would have reduced the cost per live birth.

### Pregnancy- and delivery-related costs

The cost estimation in our study also encompassed pregnancy-related expenses of prenatal care with ultrasound examinations, caesarean section delivery, and hospital stays for both the mother and baby post-delivery. It is noteworthy that in all cases of births following UTx, the mode of delivery was via caesarean section rather than vaginal delivery. This choice of delivery mode elevates the childbirth costs compared to those in a common population with mostly vaginal delivery. The rationale behind the exclusive use of caesarean section as the mode of delivery after UTx is multifaceted. Uterus-transplanted women typically experience some degree of fibrotic stenosis along the vaginal–vaginal anastomosis line and this site is characterized by very low elasticity. Additionally, women with MRKH typically have an unphysiological neovagina, which may further complicate vaginal delivery. Moreover, uncertainties persist regarding the contraction pattern of the denervated uterine graft during labour. Taken together, these factors strongly discourage the option of vaginal delivery.

The expenses for antenatal care, delivery, and postnatal care for the children in this study were significant, exceeding €130 000 in total. On a per-child basis, this equates to €14 706. One might argue that the expenses associated with pregnancy examinations, delivery, and subsequent care for both mother and child are standard costs incurred during childbirth and, therefore, should not be factored into the analysis. In fact, these expenses were omitted from the theoretical estimation of UTx costs in the Netherlands, which aimed to assess the feasibility of integrating UTx as an official infertility treatment in the country (Peters *et al.*, 2020).

### Possible future cost reductions

It is important to highlight that the achievement of nine live births from six women, resulting in an intention-to-treat take-home-baby rate of 6 out of 9 (67%), should be viewed as a highly satisfactory outcome for the inaugural scientific trial of such a complex infertility treatment. These favourable results can be compared to the modest results in the early days of the IVF era or the initial challenges of graft and patient survivals, faced at introduction of transplant procedures, such as liver and heart transplants. However, it is anticipated that the take-home-baby rate of UTx will continue to improve in the future.

In the coming years, there are several anticipated refinements and modifications that are likely to reduce the costs associated with clinical UTx. One significant factor in cost reduction is the improvement of surgical success, defined as achieving a viable graft with good blood flow and regular menstrual cycles. In the current study, the surgical success rate was 7 out of 9 (78%), a figure consistent with updated global data from over 70 UTx procedures, encompassing both LD and DD transplants, which showed an overall surgical success rate of 77% (Brannstrom *et al.*, 2023a). However, these and our figures include early procedures and given the substantial learning curve associated with UTx, it is expected that success rates will increase over time. Notably, the introduction of robotic-assisted UTx, incorporating robotic LD hysterectomy, has reported a surgical success rate of 88% (Brannstrom *et al.*, 2023a), albeit including initial failures during the early stages of surgical development (Brannstrom *et al.*, 2020). Based on these findings, it is plausible that current procedures conducted by experienced teams yield a surgical success rate well above 90%. Evidence from an ongoing trial in Germany, which commenced in 2017 and has thus far included four LD UTx procedures conducted via laparotomy in LD and recipient, demonstrated a 100% surgical success rate (Brucker *et al.*, 2020). A reduction in surgical failure rates would naturally lead to a higher live birth rate per UTx procedure and thereby to considerable decrease in costs per live birth.

There are other refinements of the UTx procedure that also would reduce costs. In the present study, we waited for 12 months until the first ET was performed, and this waiting time was conservative and adjusted to the recommendations (Deshpande *et al.*, 2013) for women who have undergone other types of transplants, such as kidney and liver. Women receiving kidney and liver transplants are in decreased health status when transplanted and require a recovery period of about a year, during which immunosuppressive medication doses may be gradually reduced. In contrast, recipients of UTx are generally in good health at the time of surgery and would not need a long recovery period. Studies have demonstrated that immunosuppressive regimens in UTx can be standardized without the need for initial administration of potentially teratogenic agents like mycophenolate mofetil (Brannstrom *et al.*, 2020). Thus, the time from UTx until the first ET has now been shortened to around 3–8 months (Johannesson *et al.*, 2019). This concept with early post-UTx ET will naturally reduce the time from UTx to childbirth(s), consequently diminishing the overall period necessitating expensive tacrolimus immunosuppressive therapy, having a yearly cost of around €5100 per patient in the present study.

In the present study, three women gave birth twice. It is anticipated that by employing exclusively blastocyst ET and possibly also utilizing preimplantation genetic testing for aneuploidy (PGT-A) to exclude aneuploid embryos, the duration from UTx to achieving a viable pregnancy will be shortened. Consequently, by shortening the interval between UTx and first ET as well as selecting embryos with high pregnancy potential, more women will have the opportunity to deliver two or even three babies, thereby reducing the cost per live birth following UTx. The limitation on the number of live births is not attributed to the strains on the uterus during pregnancies but rather to the duration of immunosuppression required for the recipient. Notably, a progressive decline in kidney function has been observed post-UTx, and the general recommendation advises maintaining the graft for a maximum of 6 years. In the present study, one patient who experienced two live births retained her graft for nearly 7 years, resulting in approximately a 20% decrease in kidney function (Ekberg *et al.*, 2023).

UTx can be performed both as a more costly LD procedure or as a DD procedure, wherein the uterus is obtained from a multi-organ donor alongside organs like kidneys, liver, lungs, and heart. In the case of DD UTx, donor-related expenses are minimal, comprising only standard laboratory screening costs and an additional 0.5–1 h to the procurement surgery time (Castro *et al.*, 2021). Based on the cost analysis conducted in this study, estimating LD-related expenses at around €28 000, the cost per child in DD UTx would decrease to approximately €97 000.

### Costs in a healthcare perspective

Our primary rationale for adopting a healthcare perspective is that prioritization occurs within the confines of healthcare budgets rather than at a societal level. The added cost at a societal level would mainly be the cost of productivity loss during sick leave, which was included in our previous study of the costs up to 2 months (Davidson *et al.*, 2021) and would amount to around 26% of the healthcare costs. When considering the potential introduction of a new intervention and given resource limitations, it is crucial to assess the impacts on the healthcare budget and potential displacements of other care. The development of new interventions raises questions about their integration into the healthcare system as standard care. This necessity has led to the emergence of health technology assessment (HTA) as a decision-making tool to determine the feasibility of such introductions. The assessment typically involves evaluating patient safety, efficacy, costs, cost-effectiveness, as well as ethical and socio-legal considerations related to the intervention (O'Rourke *et al.*, 2020). Publicly funded healthcare systems face challenges posed by resource constraints, necessitating prioritization, and rationing, particularly when introducing new innovations (Bhatia, 2020). The abundance of initial data from UTx trials, including their surgical success rates, has provided a solid foundation to assess both the safety and efficacy of UTx (Brannstrom *et al.*, 2023a), hence it is timely to also explore the cost and cost-effectiveness of UTx.

## Conclusion

In conclusion, this study presents the first estimation of costs per live birth in UTx, finding a total cost per child of €124 894, with over half of the costs attributed to preoperative investigations and surgeries. Cost estimations for novel medical procedures, such as UTx, are essential for discussions on affordability and prioritization within healthcare systems. Despite challenges posed by resource constraints, UTx trials worldwide have shown promising results, warranting further exploration of its cost-effectiveness. Moving forward, ongoing refinements in UTx procedures are anticipated to reduce costs and enhance outcomes, positioning UTx as a viable option for women with AUFI.

## Data Availability

The data underlying this article will be shared on reasonable request to the corresponding author.
